# Joint association of estimated glucose disposal rate and body mass index with new-onset stroke

**DOI:** 10.3389/fneur.2025.1529752

**Published:** 2025-05-09

**Authors:** Ting Yu, Da-Ming Shao, Tian Lv, Yu-Jun Xiong

**Affiliations:** ^1^Department of Neurosurgery, Tiantai People's Hospital, Zhejiang, China; ^2^Department of Rheumatology, The University of Chicago Medical Center, Chicago, IL, United States; ^3^Department of Neurology, Zhuji Affiliated Hospital of Wenzhou Medical University, Zhuji, China; ^4^Department of Gastroenterology, Beijing Hospital, National Center of Gerontology, Institute of Geriatric Medicine, Chinese Academy of Medical Sciences, Beijing, China

**Keywords:** BMI, CHARLS, eGDR, mediating effect, stroke

## Abstract

**Background:**

Stroke is a major global health concern, and understanding its modifiable risk factors is critical for prevention. Body mass index (BMI) and estimated glucose disposal rate (eGDR), indicators of adiposity and insulin sensitivity, respectively, are independently associated with stroke risk. However, the combined effects of these factors remain underexplored.

**Methods:**

This study utilized data from the China Health and Retirement Longitudinal Study (CHARLS), including 7,212 adults aged over 45 years. Cox proportional hazards models assessed the independent and joint associations of BMI and eGDR with new-onset stroke. Mediation analysis evaluated BMI’s role in the eGDR-stroke relationship. Subgroup analyses by age, sex, and BMI categories were conducted.

**Results:**

Over a 7-year follow-up, 587 participants (8.14%) experienced new-onset stroke. Higher BMI was positively associated with stroke incidence, while lower eGDR was linked to increased stroke risk. Participants with both obesity (BMI over 28 kg/m^2^) and lower eGDR faced the highest stroke risk (HR: 2.63; 95% CI: 1.78–3.89). Mediation analysis revealed that BMI significantly mediated 16.78% of the association between eGDR and new-onset stroke. Subgroup analyses showed consistent associations across age, sex, and BMI categories.

**Conclusion:**

This study highlights the significant and interconnected roles of BMI and eGDR in new-onset stroke risk, with a compounding effect observed in individuals with obesity and low eGDR. Addressing both insulin resistance and adiposity through targeted interventions could effectively reduce stroke risk, particularly in high-risk populations.

## 1 Introduction

New-onset stroke remains a critical global health issue, contributing significantly to morbidity and mortality, particularly among older adults. Stroke is a leading cause of disability-adjusted life years and the second most common cause of death worldwide (1). The Global Burden of Disease study estimates that over 12 million people suffered a first-time stroke in 2019, with low- and middle-income countries disproportionately affected (2). In China, the stroke burden is particularly severe, with stroke being the leading cause of death and disability (3). This underscores the urgent need to identify modifiable risk factors and implement targeted interventions to reduce stroke incidence and its associated health and economic burden.

Body mass index (BMI), a well-established metric of obesity, is a well-recognized risk factor for stroke (4, 5). Obesity exacerbates the risk of stroke through mechanisms such as increased blood pressure, dyslipidemia, and pro-inflammatory states, all of which impair vascular health (6, 7). While higher BMI is generally considered a risk factor for stroke, recent studies suggest a more complex, nonlinear relationship (8).

Estimated glucose disposal rate (eGDR), an indicator of insulin sensitivity, has emerged as a novel biomarker associated with metabolic health and cardiovascular risk. Insulin resistance (IR), defined as the need for higher doses of insulin to achieve a normal physiological response, has been linked to an increased risk of stroke through mechanisms such as atherosclerosis (9). Clinically, the euglycemic-hyperinsulinemic clamp is the gold standard for assessing IR, while the homeostasis model assessment of insulin resistance (HOMA-IR) serves as a widely accepted surrogate (10, 11). Due to the high cost and complexity of these methods, noninvasive alternatives, such as the estimated glucose disposal rate (eGDR), are gaining attention. Research has validated eGDR as an accurate measure of IR, with low eGDR levels significantly associated with increased mortality in individuals with diabetes (12). Additionally, eGDR has been identified as a predictor of adverse cardiovascular outcomes, including stroke (13).

However, research on the combined impact of eGDR and BMI on stroke remains limited and lacks systematic exploration. Moreover, the potential mediating role of BMI in the relationship between eGDR and stroke has not been thoroughly investigated. This study utilizes data from the China Health and Retirement Longitudinal Study (CHARLS) to investigate the associations of eGDR, BMI, and their combined effects on stroke risk, with the goal of providing insights that could inform prevention strategies tailored to high-risk populations in China.

## 2 Materials and methods

### 2.1 Study design and participants

This study is a secondary analysis of the China Health and Retirement Longitudinal Study (CHARLS), a national, population-based cohort targeting Chinese adults aged 45 and above.^1^ The sample was drawn from 150 counties or districts and 450 villages across 28 provinces in China, spanning the period from 2011 to 2020 (14).

**Figure 1:**
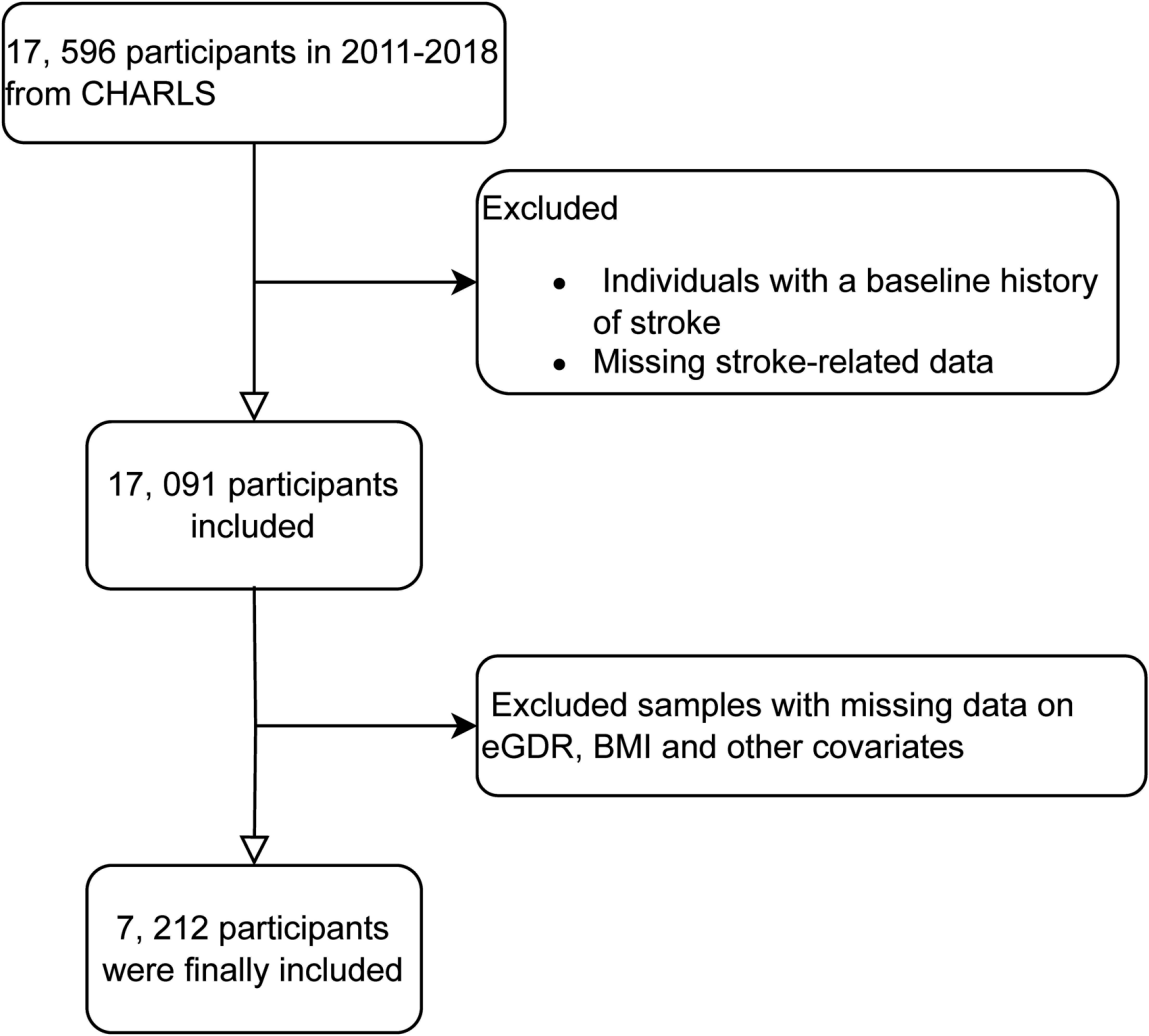
Flowchart of participant screening.

For our analysis, we utilized data from waves 1 to 4 of CHARLS, covering the period from 2011 to 2018. Data from wave 5 (conducted in 2020) was excluded due to the impact of COVID-19. Wave 1 in 2011 included 17,596 participants; individuals without baseline stroke related data or those reporting stroke at baseline were excluded. Subsequently, during follow-up from 2013 to 2018, participants with missing data on stroke related questionnaire, BMI, eGDR, or other covariates were also excluded. The exclusion criteria for this study included participants with missing data on key variables such as educational attainment, alcohol consumption status, hemoglobin levels, smoking status, diabetes mellitus diagnosis, residential status, uric acid levels, heart disease-related information, and other missing covariate data. These exclusions were necessary to ensure the integrity and completeness of the dataset, allowing for more accurate and reliable statistical analysis (Figure 1).

### 2.2 Assessment of eGDR and BMI

In the current study, estimated glucose disposal rate (eGDR, mg/kg/min) was calculated using a previously established formula: eGDR = 21.158 − (0.09 × WC) − (3.407 × HT) − (0.551 × HbA1c), where WC represents waist circumference (cm), HT is hypertension status (yes = 1, no = 0), and HbA1c is the glycated hemoglobin level (% DCCT). Waist circumference was measured at the natural waist position (11), by trained staff at the end of the participant’s normal exhalation (15). The presence of hypertension was assessed based on participants’ self-reports of whether they had been diagnosed with hypertension by a physician (16). Participants undergoing blood tests were instructed to fast overnight, and venous blood samples were collected by medically trained personnel. These samples were immediately stored at −4°C and transported to a central laboratory in Beijing for analysis within 2 weeks. The HbA1c assay was performed using the boronate affinity high performance liquid chromatography (HPLC) method (17). BMI was calculated as weight (kg) divided by height squared (m^2^).

### 2.3 Assessment of new-onset stroke and their follow-up time

The primary outcome of this study is the occurrence of stroke. Stroke cases were identified based on the question, “Have you been diagnosed with a stroke?” Participants who responded “yes” were classified as stroke patients (18). The onset of stroke was recorded as the time of initial diagnosis.

The occurrence of stroke was calculated in different cases. For participants who did not report stroke at their most recent follow-up, the event timing was determined as the difference between the year of the last survey and the baseline year. For those who did develop stroke, the timing was based on the difference between the earliest reported year of stroke onset and the baseline year (19).

### 2.4 Covariate

According to prior research and clinical experts, potentially confounding and modifying variables were identified as follow: age, sex (male or female), residence (urban, rural), education (less than high school, high school, college). Clinical indicators such as uric acid, hemoglobin, blood lipids and glucose were measured in the laboratory. Heart disease, dyslipidemia and diabetes mellitus were evaluated through a standardized questionnaire that inquired whether participants had ever been diagnosed by a doctor with these conditions (20). Alcohol drinking status was classified into two distinct categories as ever/present or never. Smoke status was defined as former smoke but now quit, still smoke and never smoke (21, 22).

### 2.5 Statistical analysis

Data were presented as means and standard deviations (SDs) for continuous variables with normal distributions and as medians with interquartile ranges for those that were non-normally distributed. Categorical variables were described as frequencies with percentages. Baseline characteristics between groups were compared using the chi-squared test, analysis of variance (ANOVA), or the Kruskal–Wallis rank-sum test, depending on the type of data (23).

We calculated the follow-up person-time for each participant, starting from the baseline survey (2011–2012) until either the date of stroke diagnosis or the end of follow-up (2017–2018), whichever occurred first. Cox proportional hazard regression models were used to estimate hazard ratios (HRs) and 95% confidence intervals (CIs) for outcomes associated with BMI and eGDR. Three models were developed: Unadjusted model as Model 0; Model 1 adjusted for age, sex, education, smoking, and alcohol consumption; Model 2 included the adjustments from Model 1 plus diabetes history, uric acid, dyslipidemia, hemoglobin, and residence. We also used 3-knot restricted cubic spline (RCS) regression to explore potential nonlinear associations.

**Figure 2:**
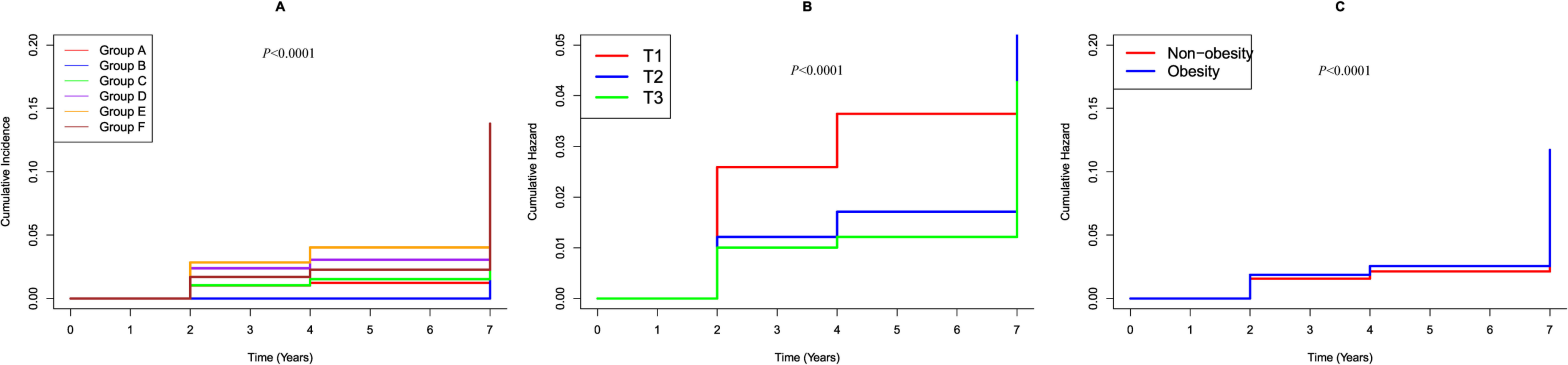
K–M plot of stroke by BMI and eGDR subgroups. **(A)** Categorized by joint variable of BMI and eGDR; Group A, 10.842 mg/kg/min < eGDR ≤ 17.812 mg/kg/min and non-obesity; Group B, 10.842 mg/kg/min < eGDR ≤ 17.812 mg/kg/min and obesity; Group C, 8.031 mg/kg/min < eGDR ≤10.842 mg/kg/min and non-obesity; Group D, 8.031 mg/kg/min < eGDR ≤ 10.842 mg/kg/min and obesity; Group E, 1.129 mg/kg/min ≤ eGDR ≤ 8.031 mg/kg/min and non-obesity; Group F, 1.129 mg/kg/min ≤ eGDR ≤ 8.031 mg/kg/min and obesity. **(B)** Categorized by eGDR; T1, 1.129 mg/kg/min ≤ eGDR≤8.031 mg/kg/min; T2, 8.031 mg/kg/min < eGDR ≤10.842 mg/kg/min; T3, 10.842 mg/kg/min < eGDR≤17.812 mg/kg/min. **(C)** Categorized by BMI; Obesity: BMI ≥ 28 kg/m^2^; Non-obesity: BMI < 28 kg/m^2^.

To evaluate the combined effects on stroke, participants were stratified into six groups based on their BMI (<28 kg/m^2^ defined as non-obesity and ≥28 kg/m^2^ as obesity) (24) and eGDR (categorized as three groups) (13, 25). In these groups, hazard ratios (HRs) for stroke incidence were calculated, using individuals with 1.129 mg/kg/min ≤ eGDR≤8.031 mg/kg/min and non-obesity in quartile 1 as the reference group. We used the Kaplan–Meier survival curve to estimate the median stroke-free survival time of the population (Figure 2) and conducted a multivariable Cox regression analysis to examine associated risk factors (Table 1).

**Table 1:** Risk classification of new-onset stroke based on BMI and eGDR by multiple cox regression analysis.

	Model 0	Model 1^a^	Model 2^b^
BMI	1.05 (1.03,1.07)^***^	1.06 (1.04,1.08) ^***^	1.04 (1.02,1.06)^***^
Non-obesity	ref	ref	ref
Obesity	1.56 (1.26,1.94)^***^	1.73 (1.39,2.15)^***^	1.33 (1.06,1.67)^*^
eGDR	0.80 (0.77,0.82)^***^	0.81 (0.78,0.85)^***^	0.84 (0.80,0.87)^***^
T1	ref	ref	ref
T2	0.49 (0.41,0.59)^***^	0.55 (0.45,0.66)^***^	0.60 (0.49,0.73)^***^
T3	0.30 (0.24,0.38)^***^	0.37 (0.29,0.47)^***^	0.41 (0.32,0.52)^***^
Joint variable			
Q1	ref	ref	ref
Q2	0.72 (0.10,5.17)	0.82 (0.11,5.85)	0.7 (0.10,5.06)
Q3	1.54 (1.19,1.99)^***^	1.50 (1.16,1.95)^**^	1.42 (1.10,1.84)^**^
Q4	2.11 (1.37,3.25)^***^	2.39 (1.55,3.69)^***^	2.15 (1.39,3.33)^***^
Q5	3.26 (2.58,4.11)^***^	2.91 (2.30,3.68)^***^	2.55 (2.00,3.24)^***^
Q6	3.36 (2.49,4.54)^***^	3.4 (2.51,4.61)^***^	2.48 (1.79,3.42)^***^

^a^Model 1 adjusted for age, sex, education, smoke, alcohol drink. ^b^Model 2 adjusted for age, sex, education, smoke, alcohol drink, diabetes mellitus, uric acid, dyslipidemia, hemoglobin, residence. ^*^p < 0.05, ^**^p < 0.01, ^***^p < 0.001. T1, 1.129 mg/kg/min ≤ eGDR ≤ 8.031 mg/kg/min; T2, 8.031 mg/kg/min < eGDR ≤ 10.842 mg/kg/min; T3, 10.842 mg/kg/min < eGDR ≤ 17.812 mg/kg/min; Q1, 10.842 mg/kg/min < eGDR ≤ 17.812 mg/kg/min and non-obesity; Q2, 10.842 mg/kg/min < eGDR ≤ 17.812 mg/kg/min and obesity; Q3, 8.031 mg/kg/min < eGDR ≤ 10.842 mg/kg/min and non-obesity; Q4, 8.031 mg/kg/min < eGDR ≤ 10.842 mg/kg/min and obesity; Q5, 1.129 mg/kg/min ≤ eGDR ≤ 8.031 mg/kg/min and non-obesity; Q6, 1.129 mg/kg/min ≤ eGDR ≤ 8.031 mg/kg/min and obesity.

A mediation analysis was conducted to evaluate the direct and indirect effects between eGDR and stroke through elevated BMI. To determine whether the associations between eGDR and the risk of stroke varied by demographic characteristics, we assessed potential effect modification by age (<60 vs. ≥60 years), sex (women vs. men), and BMI (≥28 vs. <28). To perform sensitivity analysis, comparison between included and excluded participants and multiple imputations for missing variables were performed in Supplementary Tables 1–3. All statistical analyses were performed using R software (version 4.2.1). Multiple imputations were conducted with the “charlsR” package. Mediation analysis utilized the “mediation” package, while Cox regression was carried out using the “survival” package. A two-sided *p*-value of <0.05 was considered statistically significant (26).

## 3 Results

### 3.1 Study participants and baseline characteristics

The final cohort consisted of 7, 212 adults, of whom 587 were identified as having new-onset stroke (Table 2). The mean age was 58.14 ± 8.97 years, with males comprising 44.38% of the sample. In the group with new-onset stroke, participants were more likely to be older, have a higher BMI, and exhibit a greater prevalence of heart disease. Additionally, this group demonstrated higher levels of blood glucose, a higher prevalence of diabetes, elevated blood lipid levels and dyslipidemia, as well as increased creatinine and uric acid levels. Furthermore, they showed lower eGDR compared to those without stroke.

**Table 2:** Baseline characteristics of participants.

	Overall (*n* = 7, 212)	No stroke (*n* = 6, 625)	Stroke (*n* = 587)	*p* value
Age (years)	58.14 ± 8.97	57.88 ± 8.94	61.11 ± 8.71	<0.0001
Sex (Male %)	3,201 (44.38)	2,919 (44.06)	282 (48.04)	0.07
BMI (kg/m^2^)	23.63 ± 3.87	23.55 ± 3.86	24.46 ± 3.95	<0.0001
Hemoglobin (g/dL)	14.35 ± 2.19	14.33 ± 2.18	14.53 ± 2.26	0.04
Education (%)				0.28
Less Than High School	6,510 (90.27)	5,975 (90.19)	535 (91.14)	
College	86 (1.19)	83 (1.25)	3 (0.51)	
High School	616 (8.54)	567 (8.56)	49 (8.35)	
Residence				0.49
Rural	4,841 (67.12)	4,455 (67.25)	386 (65.76)	
Urban	2,371 (32.88)	2,170 (32.75)	201 (34.24)	
Glucose (mg/dL)	109.27 ± 33.87	108.73 ± 33.17	115.42 ± 40.55	<0.001
Creatinine (mg/dL)	0.77 ± 0.18	0.77 ± 0.18	0.80 ± 0.19	<0.001
Uric acid (mg/dL)	4.39 ± 1.22	4.38 ± 1.21	4.56 ± 1.30	<0.001
Dyslipidemia (yes%)	630 (8.74)	515 (7.77)	115 (19.59)	<0.0001
TC (mg/dL)	193.51 ± 38.08	193.12 ± 38.01	197.96 ± 38.56	<0.01
HDL-C (mg/dL)	51.30 ± 15.19	51.54 ± 15.19	48.60 ± 14.92	<0.0001
LDL-C (mg/dL)	116.58 ± 34.75	116.33 ± 34.53	119.41 ± 37.05	0.05
TG (mg/dL)	131.04 ± 94.48	129.46 ± 92.61	148.96 ± 111.97	<0.0001
Smoke status (%)				<0.01
Former, now quit	571 (7.92)	504 (7.61)	67 (11.41)	
Never	4,509 (62.52)	4,168 (62.91)	341 (58.09)	
Current	2,132 (29.56)	1,953 (29.48)	179 (30.49)	
Alcohol drink (%)				0.47
No	4,873 (67.57)	4,468 (67.44)	405 (68.99)	
Yes	2,339 (32.43)	2,157 (32.56)	182 (31.01)	
Diabetes Mellitus (%)	373 (5.17)	305 (4.60)	68 (11.58)	<0.0001
Heart disease = yes (%)	749 (10.39)	630 (9.51)	119 (20.27)	<0.0001
eGDR	9.36 ± 2.28	9.46 ± 2.25	8.19 ± 2.32	<0.0001
Follow up time (years)	6.90 ± 0.67	7.00 ± 0.00	5.80 ± 2.03	<0.0001

BMI, body mass index; TC, total cholesterol; HDL, high-density lipoprotein; LDL, low-density lipoprotein; TG, triglycerides; eGDR, estimated glucose disposal rate.

### 3.2 Correlation between eGDR, BMI, and new-onset stroke

**Figure 3:**
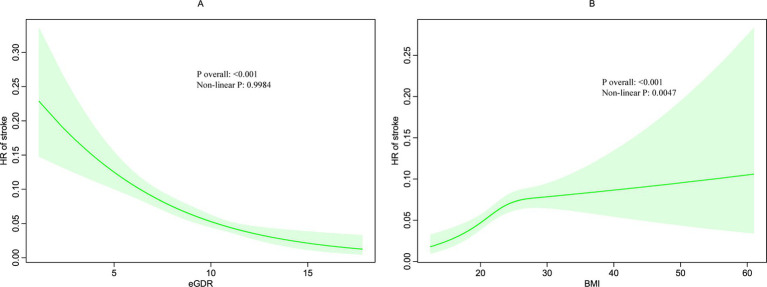
Restricted cubic spline (RCS) for the association between BMI **(B)** and eGDR **(A)** with the risks of stroke.

The relationships between these factors and new-onset stroke were further examined using RCS curves, illustrated in Figures 3A,B. The RCS analysis demonstrated that the eGDR, treated as a continuous variable, was significantly associated with a decreased adjusted linear risk of stroke (*P* overall < 0.001). Furthermore, the relationship between BMI and stroke risk was significant but nonlinear (*P* overall < 0.001, non-linear *p* = 0.0047). Notably, a BMI below 23.23 was positively associated with stroke risk (*P* overall = 0.005), but this association plateaued above 23.23, suggesting a threshold effect.

### 3.3 Associations of eGDR, BMI and cumulative eGDR-BMI with new-onset stroke

During the follow up, 587 participants (8.14%) developed stroke. Figure 2 shows Kaplan–Meier curves illustrating the cumulative incidence of stroke among all participants. Participants with higher eGDR demonstrated a significantly reduced risk of stroke compared to those with the lowest eGDR levels (*p* < 0.0001). Conversely, obesity was associated with a markedly increased risk of stroke relative to individuals with lower BMI (*p* < 0.0001). Notably, the combined presence of low eGDR and obesity was linked to a substantially higher risk of stroke compared to participants with high eGDR and non-obese status (*p* < 0.0001).

A multivariable cox regression analysis was then performed to assess the relationship between eGDR, BMI and their combined effect on stroke, as detailed in Table 1. BMI, when analyzed as a continuous variable, was found to be associated with an elevated risk of stroke. Conversely, eGDR showed a negative association with stroke, indicating that low eGDR was linked to an increased risk of developing stroke. Initially, in the baseline model (Model 0), individuals in the obesity group had a significantly higher risk of developing stroke compared to the non-obesity group (*p* < 0.001). These associations remained significant after adjusting for age, sex, education, smoke, alcohol drink, diabetes mellitus, uric acid, dyslipidemia, hemoglobin, residence.

Similarly, when eGDR was divided into three groups in the Model 0, participants in the T3 group exhibited a significantly lower risk of stroke compared to those in the T1 group (*p* < 0.001). These significant associations persisted after adjusting for covariates in subsequent models (Model 1 and 2).

A joint analysis was performed to examine the combined impact of eGDR and BMI on the risk of stroke. The findings indicated that individuals with both low eGDR and obesity faced the highest risk of stroke (Table 1). Notably, participants with eGDR levels between 10.842 and 17.812 mg/kg/min and obesity had hazard ratios (HRs) of 0.70 (95% CI: 0.10–5.06) for stroke compared to those within the same eGDR range but without obesity, even after adjusting for covariates. In contrast, all other combinations of eGDR and obesity status showed statistically significant associations with stroke risk.

### 3.4 Mediation analyses of eGDR of BMI on stroke

**Figure 4:**
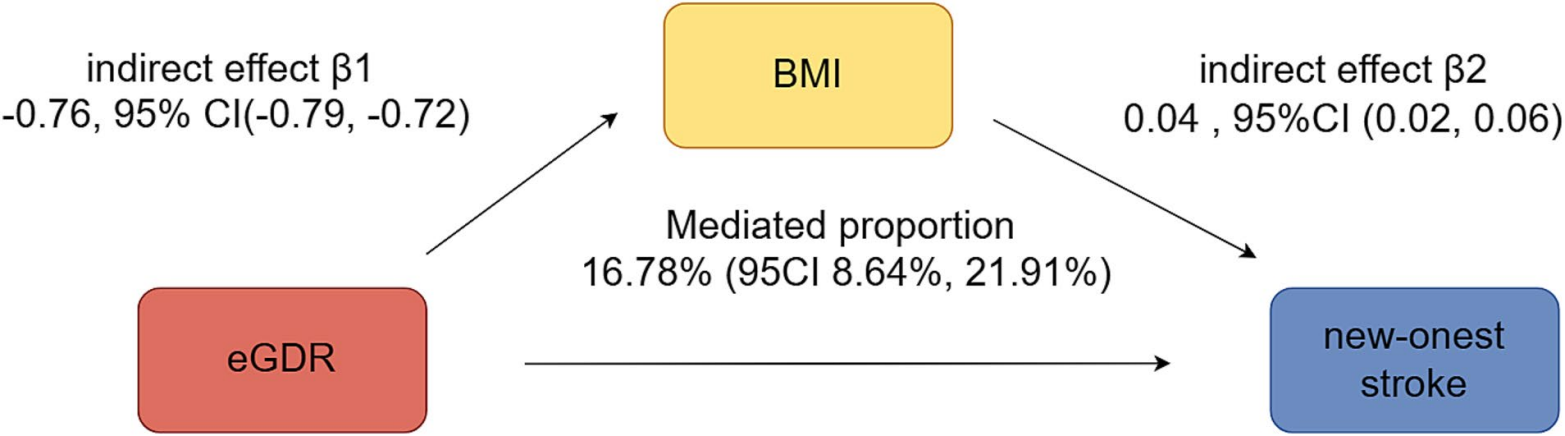
Mediation analyses of BMI and eGDR on new-onset stroke.

Figure 4 illustrated the mediation effects between eGDR, BMI, and new-onset stroke. BMI significantly mediated 16.78% (95% CI: 8.64–21.91%) of the association between eGDR and new-onset stroke.

### 3.5 Subgroup analyses

**Figure 5:**
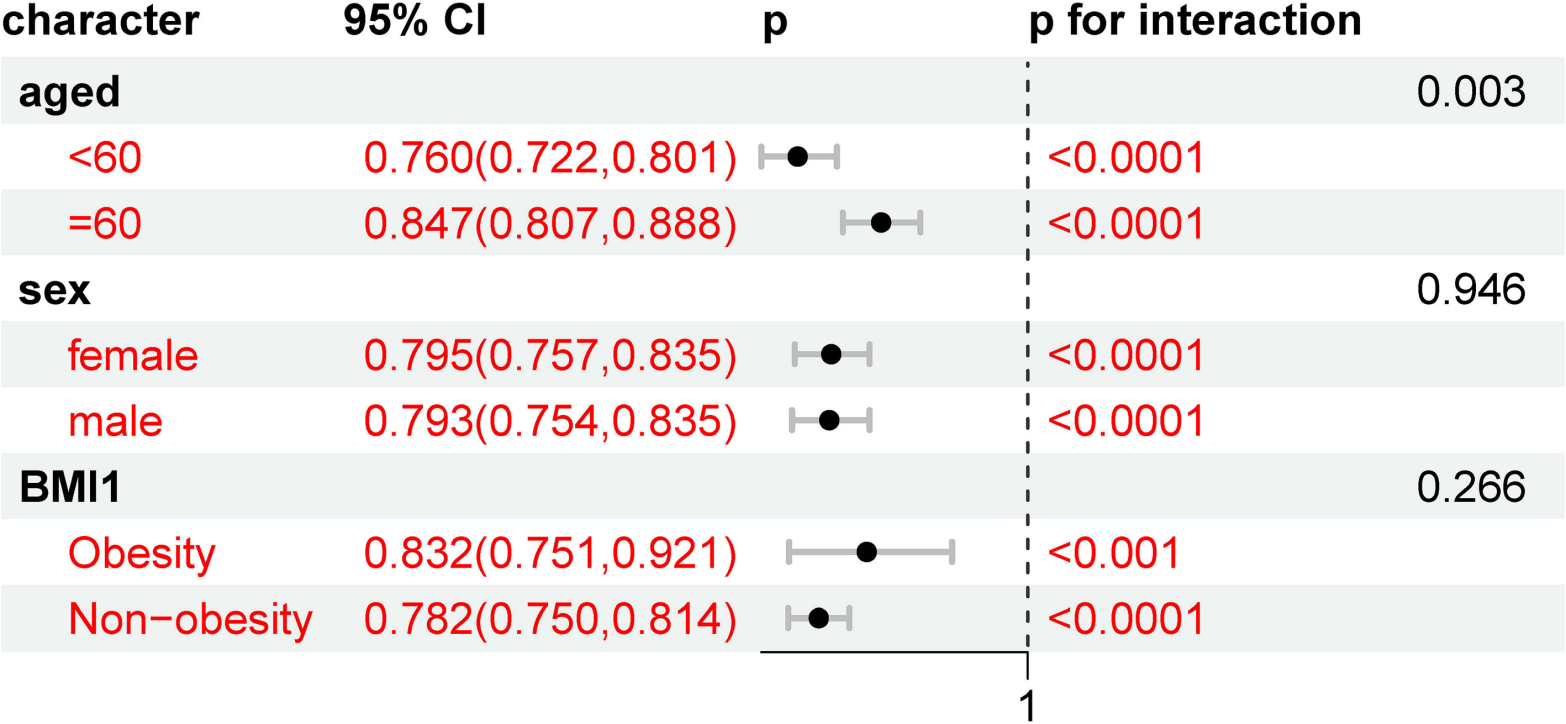
Subgroup analyses of eGDR on new-onset stroke with different demographics.

The results of the subgroup analyses are presented in Figure 5. When stratified by demographics, including age (over 60 vs. under 60), obesity status (obesity vs. non-obesity), and sex (female vs. male), all of these subgroups showed a statistically significant association with new-onset stroke. This suggests that the risk of new-onset stroke was not influenced by these demographic variables in this analysis.

### 3.6 Sensitivity analyses

In the sensitivity analyses, we compared the baseline characteristics of excluded and included participants, as presented in Supplementary Table 1. The overall cohort consisted of 17,526 individuals, with 10,314 excluded and 7,212 included in the final analysis. Significant differences were observed between the two groups in variables such as BMI, hemoglobin levels, education, residence, glucose levels, and the prevalence of dyslipidemia, diabetes mellitus, and heart disease (all *p* < 0.0001). However, no significant differences were found in age, sex, or smoking status, suggesting that the exclusion criteria did not introduce major biases in these demographic factors.

The baseline characteristics of the final cohort after multiple imputation for missing variables were summarized in Supplementary Table 2. The cohort included 7, 998 adults, of whom 655 were identified with new-onset stroke. Participants with stroke were older, had higher BMI, and exhibited a greater prevalence of comorbidities such as heart disease, diabetes, and dyslipidemia compared to those without stroke. Additionally, they had elevated levels of blood glucose, creatinine, and uric acid, along with lower eGDR. These findings were consistent with the pre-imputation results, indicating that the multiple imputation process did not substantially alter the observed associations, thus supporting the robustness of our data.

Supplementary Table 3 details the risk classification of new-onset stroke based on BMI and eGDR using multiple Cox regression analysis after multiple imputation. BMI, as a continuous variable, was positively associated with stroke risk, whereas eGDR showed a negative association. The joint analysis revealed that individuals with both low eGDR and obesity had the highest risk of stroke. These associations remained significant after adjusting for covariates such as age, sex, education, smoking, alcohol consumption, diabetes, uric acid, dyslipidemia, hemoglobin, and residence. The consistency of these results across models, even after multiple imputation, underscores the reliability of our findings and reinforces the importance of considering both BMI and eGDR in stroke risk assessment.

## 4 Discussion

Our study suggests that both BMI and eGDR independently contribute to new-onest stroke risk, with a compounding effect observed in individuals with both low eGDR and obesity. BMI, a well-established marker of adiposity, has been widely recognized as a significant risk factor for stroke, potentially through its influence on atherosclerosis, hypertension, and systemic inflammation. Conversely, eGDR, a surrogate marker of insulin sensitivity, was negatively associated with stroke risk in our study, highlighting its protective role. Importantly, our analysis demonstrated that BMI mediated a significant proportion of the relationship between eGDR and new-onset stroke, underscoring the intricate interplay between these variables.

The positive association between BMI and stroke has been supported by extensive research. Adiposity, as reflected by higher BMI, contributes to pro-inflammatory states, metabolic dysregulation, and increased atherosclerosis, all of which elevate the risk of cerebrovascular events (23). However, our study revealed that the positive association between BMI and stroke incidence was observed more significantly among participants with a BMI of 23.23 kg/m^2^ or lower, consistent with findings from previous research (27). For example, Iona et al. (28) reported positive log-linear associations between both measured and genetically predicted BMI and stroke risk in the relatively lean Chinese population. Nevertheless, whether the observed U- or J-shaped associations between BMI and mortality reflect a true causal relationship or are influenced by uncontrolled reverse causality remains uncertain. Our findings align with and further contextualize these observations, highlighting the intricate relationship between BMI, metabolic health, and stroke outcomes.

Insulin resistance is a pivotal mechanism contributing to the heightened risk of cardiovascular disease. It fosters atherogenic lipid profiles by increasing very low-density lipoprotein particles, which are metabolized into remnant lipoproteins that promote atherosclerosis (29). Furthermore, insulin resistance induces pro-inflammatory and procoagulant states, amplifying atherosclerotic processes (30). Previous studies have consistently linked insulin resistance to diabetes, dyslipidemia, and hypertension—key risk factors for stroke (31). A longitudinal study involving 1,476 participants established a linear relationship between eGDR and stroke incidence in individuals with type 2 diabetes, with higher eGDR levels associated with reduced stroke risk (12). Consistent with this, our study identified individuals with both low eGDR and obesity as having the highest stroke risk. This finding suggests a synergistic effect, where the combined impact of insulin resistance and excessive adiposity exacerbates cerebrovascular vulnerability. The mediating role of BMI in the eGDR-stroke relationship underscores the significant influence of obesity in modulating this association. These insights highlight the potential benefits of targeted interventions, such as weight reduction strategies, to mitigate the elevated stroke risk associated with low eGDR. An important consideration is the potential for reverse causality in our mediation analysis. While lower eGDR reflects insulin resistance, it may also contribute to increased fat storage and reduced energy expenditure, leading to weight gain and a higher BMI (32, 33). This bidirectional interaction could inflate the estimated mediation effect of BMI. Future studies using longitudinal designs or Mendelian randomization could help clarify the directionality of this relationship.

The pathophysiological mechanisms linking BMI, eGDR, and stroke risk remain complex. Adiposity is associated with insulin resistance, pro-inflammatory cytokine release, and endothelial impairment (34), while low eGDR reflects diminished glucose uptake and metabolic inefficiency (35). These overlapping pathways likely contribute to a shared biological mechanism that increases stroke susceptibility. Further investigation into these interactions is warranted, particularly studies focusing on biomarkers of inflammation and endothelial function, as well as neuroimaging studies assessing cerebral vascular changes in populations with varying BMI and eGDR levels.

Our study highlights important clinical applications and future directions in stroke prevention. Traditionally, BMI has been a key factor in assessing stroke risk, but our findings suggest that eGDR, as a marker of insulin sensitivity, should also be incorporated into routine health screenings. Relying solely on BMI may overlook individuals with metabolic dysfunction who are at elevated risk, despite having a normal BMI. Therefore, comprehensive risk assessments should include eGDR-related factors, such as waist circumference, hypertension status, and HbA1c levels, to better identify high-risk individuals. This has significant implications for clinical practice, as integrating eGDR into stroke risk stratification could enable earlier interventions, personalized lifestyle modifications, and targeted therapies to improve metabolic health. Future research should explore the feasibility of incorporating eGDR into routine health check-ups and validate its predictive value in diverse populations. Public health policies should also promote metabolic screenings in at-risk individuals, ensuring that stroke prevention strategies go beyond weight management to address underlying insulin resistance and vascular dysfunction.

Our study has several strengths, including the use of a large, nationally representative cohort with robust longitudinal data, allowing us to evaluate the independent and combined effects of BMI and eGDR on stroke risk. Additionally, the mediation analysis provides novel insights into the intermediary role of BMI in this association. However, there are limitations to consider. First, the findings are based on a Chinese cohort and may not be directly generalizable to other populations. Second, the diagnosis of stroke in surveys is primarily based on self-reported questionnaires rather than laboratory examinations. Third, the observational design limits causal inference, and further interventional studies are needed to validate our findings. Additionally, the absence of statistical testing for potential reverse causality is a limitation. Given that insulin resistance may contribute to weight gain, the estimated mediation effect of BMI could be inflated. Future studies should incorporate causal inference approaches to better. Finally, some covariates, including heart disease, dyslipidemia, diabetes mellitus, alcohol consumption, and smoking status, were selected by questionnaires, which would provoke recall and selection bias.

Future research should aim to elucidate the underlying biological mechanisms linking BMI, eGDR, and stroke. Studies incorporating biochemical markers of inflammation, adipokines, and vascular health, alongside neuroimaging techniques, could provide deeper insights into these relationships. Additionally, randomized controlled trials evaluating interventions aimed at improving insulin sensitivity and reducing obesity could inform targeted prevention strategies for stroke. By addressing these modifiable risk factors, we can advance the development of effective, multifaceted approaches to mitigate stroke risk, particularly in populations at elevated metabolic and cardiovascular risk.

## 5 Conclusion

In conclusion, current evidence highlights the significant and interconnected roles of BMI and eGDR in the development of new-onset stroke. Higher BMI is positively associated with an increased risk of new-onest stroke, while lower eGDR, indicative of reduced insulin sensitivity, demonstrates a negative association with stroke risk. Individuals with both low eGDR and obesity face the highest risk. Furthermore, BMI significantly mediates 16.78% of the relationship between eGDR and new-onset stroke. These findings underscore the importance of addressing both insulin resistance and obesity in the prevention and management of stroke. Future research, particularly prospective longitudinal studies, is needed to explore these underlying mechanisms further and to develop comprehensive, targeted strategies to mitigate new-onest stroke risk effectively.

## Data Availability

The original contributions presented in the study are included in the article/Supplementary material, further inquiries can be directed to the corresponding author.

## Footnotes

^1^ http://charls.pku.edu.cn/
