# Supplementary material for: Joint association of estimated glucose disposal rate and body mass index with new-onset stroke
Source: Front Neurol. 2025 May 9;16:1529752. doi: 10.3389/fneur.2025.1529752 (PMC12098433; doi:10.3389/fneur.2025.1529752)
Supplement: Supplementary file 1 [file Table_1.docx]

Supplementary Table 1 Baseline characteristics of exclude and included participants.

|  | Overall  (n=17, 526) | *Excluded*  *(n=10, 314)* | *Included*  *(n=7, 212)* | *P* value |
| --- | --- | --- | --- | --- |
| Age (years) | 58.98 ± 10.15 | 58.99 ± 10.20 | 58.96 ± 10.06 | 0.87 |
| Sex (Male %) | 8386(47.89) | 4927(47.82) | 3459(47.99) | 0.84 |
| BMI (kg/m^2^) | 23.47 ± 3.89 | 23.16 ± 3.80 | 23.94 ± 3.99 | <0.0001 |
| Hemoglobin (g/dL) | 14.39 ± 2.22 | 14.51 ± 2.39 | 14.22 ± 1.95 | <0.0001 |
| Education (%) |  |  |  | <0.0001 |
| Less Than High School | 15282(87.32) | 9195(89.28) | 6087(84.52) |  |
| College | 424 (2.42) | 196 (1.90) | 228 (3.17) |  |
| High School | 1795(10.26) | 908 (8.82) | 887(12.32) |  |
| Residence |  |  |  | <0.0001 |
| Rural | 10458(59.68) | 6520(63.22) | 3938(54.61) |  |
| Urban | 7066(40.32) | 3793(36.78) | 3273(45.39) |  |
| Glucose (mg/dL) | 110.32 ± 37.42 | 111.10 ± 38.98 | 109.25 ± 35.14 | <0.01 |
| Creatinine (mg/dL) | 0.78 ± 0.24 | 0.79 ± 0.23 | 0.78 ± 0.24 | <0.01 |
| Uric acid (mg/dL) | 4.46 ± 1.27 | 4.49 ± 1.27 | 4.43 ± 1.27 | 0.01 |
| Dyslipidemia (yes%) | 1612( 9.38) | 803( 7.93) | 809(11.47) | <0.0001 |
| TC (mg/dL) | 193.02 ± 38.91 | 194.19 ± 39.57 | 191.40 ± 37.93 | <0.001 |
| HDL-C (mg/dL) | 50.85 ± 15.34 | 51.80 ± 15.74 | 49.56 ± 14.69 | <0.0001 |
| LDL-C (mg/dL) | 116.05 ± 34.93 | 116.54 ± 35.36 | 115.37 ± 34.32 | 0.07 |
| TG (mg/dL) | 134.87 ± 110.32 | 132.87 ± 110.48 | 137.61 ± 110.05 | 0.02 |
| Smoke status (%) |  |  |  | 0.41 |
| Former, now quit | 2059(11.76) | 1193(11.58) | 866(12.02) |  |
| Never | 10597(60.54) | 6277(60.94) | 4320(59.98) |  |
| Current | 4847(27.69) | 2830(27.48) | 2017(28.00) |  |
| Alcohol drink (%) |  |  |  | 0.59 |
| No | 11750(67.15) | 6932(67.31) | 4818(66.92) |  |
| Yes | 5748(32.85) | 3366(32.69) | 2382(33.08) |  |
| Diabetes Mellitus (%) | 993 (5.71) | 509 (4.97) | 484 (6.78) | <0.0001 |
| Heart disease = yes (%) | 2047 (11.73) | 961 (9.35) | 1086 (15.14) | <0.0001 |
| eGDR | 9.28 ± 2.31 | 9.38 ± 2.25 | 9.13 ± 2.39 | <0.0001 |

Abbreviation: body mass index (BMI), total cholesterol (TC), high-density lipoprotein (HDL), low-density lipoprotein (LDL), triglycerides (TG), estimated glucose disposal rate (eGDR).

Supplementary Table 2 Baseline characteristics of participants after multiple imputation for missing variables.

|  | Overall  (n= 7, 998) | *No Stroke*  (n= 7, 343) | *Stroke*  (n= 655) | *P* value |
| --- | --- | --- | --- | --- |
| Age (years) | 58.20 ± 9.03 | 57.93 ± 8.98 | 61.23 ± 8.99 | <0.0001 |
| Sex (Male %) | 3528(44.11) | 3215(43.78) | 313(47.79) | 0.05 |
| BMI (kg/m^2^) | 23.64 ± 3.87 | 23.57 ± 3.86 | 24.44 ± 3.92 | <0.0001 |
| Hemoglobin (g/dL) | 14.35 ± 2.18 | 14.34 ± 2.17 | 14.49 ± 2.23 | 0.09 |
| Education (%) |  |  |  | 0.22 |
| Less Than High School | 7224(90.32) | 6628(90.26) | 596(90.99) |  |
| College | 92 (1.15) | 89 (1.21) | 3 (0.46) |  |
| High School | 682 (8.53) | 626 (8.53) | 56 (8.55) |  |
| Residence |  |  |  | 0.61 |
| Rural | 5390(67.39) | 4955(67.48) | 435(66.41) |  |
| Urban | 2608(32.61) | 2388(32.52) | 220(33.59) |  |
| Glucose (mg/dL) | 109.48 ± 34.59 | 108.94 ± 33.95 | 115.48 ± 40.67 | <0.0001 |
| Creatinine (mg/dL) | 0.77 ± 0.18 | 0.77 ± 0.18 | 0.80 ± 0.18 | <0.0001 |
| Uric acid (mg/dL) | 4.39 ± 1.21 | 4.38 ± 1.20 | 4.53 ± 1.29 | <0.01 |
| Dyslipidemia (yes%) | 699 (8.74) | 573 (7.80) | 126 (19.24) | <0.0001 |
| TC (mg/dL) | 193.59 ± 38.57 | 193.25 ± 38.58 | 197.29 ± 38.30 | <0.01 |
| HDL-C (mg/dL) | 51.17 ± 15.16 | 51.39 ± 15.18 | 48.72 ± 14.74 | <0.0001 |
| LDL-C (mg/dL) | 116.42 ± 34.65 | 116.20 ± 34.45 | 118.96 ± 36.77 | 0.06 |
| TG (mg/dL) | 133.61 ± 110.59 | 132.37 ± 110.45 | 147.61 ± 111.25 | <0.001 |
| Smoke status (%) |  |  |  | <0.01 |
| Former, now quit | 641 (8.01) | 569 (7.75) | 72 (10.99) |  |
| Never | 5012(62.67) | 4629(63.04) | 383(58.47) |  |
| Current | 2345(29.32) | 2145(29.21) | 200(30.53) |  |
| Alcohol drink (%) |  |  |  | 0.70 |
| No | 5410(67.64) | 4962(67.57) | 448(68.40) |  |
| Yes | 2588(32.36) | 2381(32.43) | 207(31.60) |  |
| Diabetes Mellitus (%) | 419 (5.24) | 344 (4.68) | 75 (11.45) | <0.0001 |
| Heart disease = yes (%) | 831(10.39) | 702 (9.56) | 129 (19.69) | <0.0001 |
| eGDR | 9.37 ± 2.29 | 9.47 ± 2.26 | 8.22 ± 2.32 | <0.0001 |
| Follow up time (years) | 6.89 ± 0.70 | 7.00 ± 0.00 | 5.70 ± 2.10 | <0.0001 |

Abbreviation: body mass index (BMI), total cholesterol (TC), high-density lipoprotein (HDL), low-density lipoprotein (LDL), triglycerides (TG), estimated glucose disposal rate (eGDR).

Supplementary Table 3 Risk classification of new-onset stroke based on BMI and eGDR by Multiple Cox Regression analysis after multiple imputation for missing variables.

| BMI | Model 0  1.05(1.03,1.07) *** | Model 1^a^  1.06(1.04,1.08) *** | Model 2^b^  1.04(1.02,1.06) *** |
| --- | --- | --- | --- |
| Non-obesity | ref | ref | ref |
| Obesity | 1.57(1.29,1.93) *** | 1.75(1.42,2.15) *** | 1.40 (1.13,1.74) ** |
| eGDR | 0.80 (0.77,0.83) *** | 0.82 (0.79,0.85) *** | 0.84 (0.81,0.87) *** |
| T1 | ref | ref | ref |
| T2 | 0.48(0.40,0.57) *** | 0.53(0.44,0.64) *** | 0.58(0.48,0.70) *** |
| T3 | 0.32(0.26,0.39) *** | 0.39(0.31,0.49) *** | 0.42(0.33,0.53) *** |
| Joint variable |  |  |  |
| Q1 | ref | ref | ref |
| Q2 | 0.61 (0.09,4.37) | 0.70 (0.10,5.00) | 0.64 (0.09,4.59) |
| Q3 | 1.42(1.12,1.81) ** | 1.39(1.09,1.78) * | 1.34(1.05,1.72) * |
| Q4 | 2.03(1.35,3.04) *** | 2.32(1.54,3.49) *** | 2.14(1.42,3.24) *** |
| Q5 | 3.11(2.51,3.86) *** | 2.79(2.24,3.47) *** | 2.50 (2.00,3.13) *** |
| Q6 | 3.27(2.47,4.32) *** | 3.33(2.51,4.42) *** | 2.60 (1.92,3.52) *** |

^a^ Model 1 adjusted for age, sex, education, smoke, alcohol drink.

^b^ Model 2 adjusted for age, sex, education, smoke, alcohol drink, diabetes mellitus, uric acid, dyslipidemia, hemoglobin, residence.

**P < 0.05, **P < 0.01, ***P < 0.001*.

T1: 1.129 mg/kg/min≤eGDR≤8.031 mg/kg/min

T2: 8.031 mg/kg/min＜eGDR≤10.842 mg/kg/min

T3: 10.842 mg/kg/min＜eGDR≤17.812 mg/kg/min

Q1: 10.842 mg/kg/min＜eGDR≤17.812 mg/kg/min and non-obesity.

Q2: 10.842 mg/kg/min＜eGDR≤17.812 mg/kg/min and obesity.

Q3: 8.031 mg/kg/min＜eGDR≤10.842 mg/kg/min and non-obesity.

Q4: 8.031 mg/kg/min＜eGDR≤10.842 mg/kg/min and obesity.

Q5: 1.129 mg/kg/min≤eGDR≤8.031 mg/kg/min and non-obesity.

Q6: 1.129 mg/kg/min≤eGDR≤8.031 mg/kg/min and obesity.
